# Care seeking for fatal illness episodes in Neonates: a population-based study in rural Bangladesh

**DOI:** 10.1186/1471-2431-11-88

**Published:** 2011-10-14

**Authors:** Hafizur R Chowdhury, Sandra C Thompson, Mohammed Ali, Nurul Alam, Mohammed Yunus, Peter K Streatfield

**Affiliations:** 1 Matlab Health Research Centre, ICDDR,B, GPO Box 128, Dhaka 1000 Bangladesh; 2 Centre for International Health, Curtin Health Innovation Research Institute, Curtin University of Technology, Australia, GPO Box U1987 Perth Western Australia 6845; 3 Combined Universities Centre for Rural Health, University of Western Australia, PO Box 109, Geraldton, Western Australia 6531, Australia; 4 Public Health Sciences Division (PHSD), ICDDR,B, GPO Box 128, Dhaka 1000 Bangladesh

## Abstract

**Background:**

Poor neonatal health is a major contributor to under-five mortality in developing countries. A major constraint to effective neonatal survival programme has been the lack of population level data in developing countries. This study investigated the consultation patterns of caregivers during neonatal fatal illness episodes in the rural Matlab sub-district of eastern Bangladesh.

**Methods:**

Neonatal deaths were identified through a population-based demographic surveillance system in Matlab ICDDR,B maternal and child health (MCH) project area and an adjoining government service area. Trained project staff administered a structured questionnaire on care seeking to mothers at home who had experienced a neonatal death. Univariate, bivariate and binary multivariate logistic regressions were performed to describe care seeking during the fatal illness episode.

**Results:**

Of the 365 deaths recorded during 2003 and 2004, 84% died in the early (0-7 days) neonatal period, with the remaining deaths occurring over the subsequent 8 to 28 days. The first resort of care by parents was a qualified doctor or paramedic in 37% of cases, followed by traditional and unqualified health care providers in 25%, while 38% sought no care. Thus, almost two thirds (63%) of neonates who died received only traditional and unqualified care or no care at all during their final illness episode. About 22% sought care from more than one provider, including 6% from 3 or more providers. Such plurality in care seeking was more likely among male infants, in the late neonatal period, and in the MCH project area.

**Conclusions:**

The high proportion of neonatal deaths that had received traditional care or no medical care in a rural area of Bangladesh highlights the need to develop community awareness about prompt medical care seeking for neonatal illnesses and to improve access to effective health care. Integration of traditional care providers into mainstream health programs should also be considered.

## Background

Understanding care seeking behaviour during serious illnesses is important in designing effective public health interventions for neonatal care. In this regard, information on care seeking during episodes of fatal illnesses would be particularly helpful. The few published studies on care seeking for neonatal illnesses in Bangladesh, a densely populated country with a very high neonatal mortality burden, have been limited to non-fatal illness episodes.

These studies have revealed a number of common harmful behaviours/practices that have important implications for neonatal survival. Major harmful practices included: various dietary restrictions for women during pregnancy and the early postpartum period; culturally mandated seclusion of women during the early postpartum period which limited health care seeking; a preference for home delivery which limited skilled birth attendance; application of crude methods such as abdominal massage for speedy birth delivery; practices such as squeezing abdomen or manual removal for speedy delivery of placenta; resuscitation of asphyxiated newborns by manipulating placenta; harmful cord care such as use of bamboo/unsterilized blade for cutting the cord and applying dirty or contaminated materials to the cord; practices that can induce hypothermia such as keeping the newborn on the ground until the placenta was born; and inappropriate feeding such as prelacteal feedings and early introduction of supplemental foods. In addition, poor knowledge, physical and social insecurity, economic insecurity and environmental insecurities were commonly cited reasons for not seeking health care during the post partum period [1-4]. In a cross-sectional survey in four rural sub districts of Bangladesh, 49% of the neonates were reported to have suffered from some kind of illnesses, for which homeopaths and unqualified village doctors comprised more than two thirds (68%) of the health provider care sought, with trained health care including government facilities sought in only 22% of such cases. Seeking care from a trained provider appeared to depend on factors such as male gender of the baby, higher birth order, father's education and income, and antenatal care attendance by the mother [5].

Few studies have looked at the role of care seeking in neonatal mortality. Mercer and colleagues in their nationwide case control study in Bangladesh found that 70% of deceased neonates had not been taken to any health care provider, or had been taken to a traditional care provider only. Surviving neonates who were controls in the study had a similarly high proportion (52%) for whom either no care or traditional health care only had been sought. The adjusted odds ratios for these risk factors were: 2.9 for use of a traditional healer for a sick newborn and 23.3 for care not being sought at all [6].

A recent study from eastern Uganda to understand why newborn babies die in low resource setting, and reported that delay in problem identification or deciding to see care (50%), delay to receive quality care at a facility (30%) and transport delay were the major contributors to newborn deaths [7].

Neonatal and maternal mortality are complementary public health priorities. As the great majority of neonatal and maternal deaths occur around delivery or immediately after delivery in the early postpartum period, health facility-based delivery is considered a key strategy to improving neonatal and maternal survival. Antenatal care, a multiple purpose strategy, is another cost-effective option for reducing neonatal and maternal morbidity and mortality [8,9]. Only 9% of deliveries in Bangladesh take place in health facilities and less than half of mothers receive one session of antenatal care, although there is a wide network of such facilities throughout the country [10,11]. However, the rate of institutional delivery and antenatal care are considerably higher in the ICDDR,B area in Matlab covering a population of 112,000, with about 27% of deliveries occurring in health facilities in 2001 [12]. This is because of the intensive maternal and child health activities carried out by ICDDR,B in the MCH project area.

The HDSS has been collecting standard health and demographic information both from the ICDDR,B serviced area (intervention area) as well as the neighbouring government serviced area (comparison area with a similar population of 112,000 [13].

When a death was identified during their monthly home visits, a health worker trained in VA interviewed the mother or a close family member within 2-6 weeks of death at the home of the deceased.

Information about care seeking can provide valuable clues about potentially harmful aspects of such care seeking behaviour which could be targeted in future programmes. However, little is known about health care seeking, particularly during the fatal neonatal illness episodes. Such information is of great importance in improving health care delivery system. At this moment, there are a large number of neonatal health programmes operating in Bangladesh. For the most part, however, their activities are not based on reliable information about the health care seeking preferences or practices of parents at the community level. The present study involved administering a structured questionnaire on care seeking to mothers/or family members at home who had experienced a neonatal death during 2003 and 2004 in the Matlab HDSS area in Bangladesh.

## Methods

### Study design and population

The International Centre for Diarrhoeal Disease Research, Bangladesh (ICDDR,B) maintains a longitudinal health and demographic surveillance system (HDSS) covering a population of'~ 224,000 people in Matlab, a rural sub-district in eastern Bangladesh. ICDDR, B provides maternal and child health services in ICDDR, B MCH-FP project area (half of HDSS area population), and the government provides medical services to population in the remaining half of the HDSS area. The ICDDR,B service delivery structure includes four community health clinics, also known as sub-centre clinics, each serving around 28,000 people. Each sub-centre clinic is staffed by two nurse-midwives and one male medical assistant. ICDDR,B also maintains a primary-care hospital at Matlab sub-district town with out-patient, in-patient and basic laboratory facilities.

The Matlab ICDDR,B hospital mainly treats children aged under-five years who present with diarrhoea, acute respiratory infection, malnutrition, and/or other common problems. It also manages the health problems of women of reproductive age. Maternity care includes birth deliveries offered through the four sub-centre clinics and the Matlab hospital. Emergency obstetric care, including caesarean section, is organized as required through collaboration and referral with the government sub-district health complex at Matlab and with secondary level hospitals at Chandpur, the district headquarters [14]**. **During household visits, the CHRWs advise pregnant women to seek antenatal care for any problem, and also encourage them to deliver at a health facility such as the ICDDR,B sub-centres or ICDDR,B Matlab hospital, regardless of whether they have any complications or not.

People from Government-serviced area receive medical services from Government health facilities. At the lowest level, the Government facilities comprise the union health centre and the family welfare centre which are analogous to the ICDDR,B sub-centre in the services provided. These are managed by government paramedics and medical assistants. There are 10 such centres in the comparison area within the surveillance area. The sub-district or upazila health complex is the primary referral centre, analogous to the ICDDR,B Matlab Hospital, with the exception that it also provides primary surgical care and essential obstetric care including caesarean sections and blood transfusion. The upazila or sub-district health complex is run by physicians, nurses, a medical assistant and other staff, and maintains good working links with the Matlab ICDDR,B Hospital, especially in terms of receiving the latter's complicated obstetric cases including those requiring surgery.

Although medical facilities and services are similar and free of costs in both areas, Government health services, as throughout Bangladesh, are plagued by scarcity of basic medical supplies and poor staff morale [15]. Female Community Health Research Workers (CHRWs) recruited from the Matlab HDSS area carry out monthly household visits in the Government service area to collect information on demographic events (birth, death, migration, abortion, marriage, divorce etc) using pre-coded forms. These data are collated and maintained in the HDSS databases [13]. This study investigated and analyzed all 365 neonatal deaths that occurred in 2003 and 2004 in the Matlab HDSS area.

### Verbal autopsy

A verbal autopsy (VA) questionnaire was developed by the Verbal Autopsy Working Group of the International Network of Field Sites with Continuous Demographic Evaluation of Populations and their Health in Developing Countries (INDEPTH; see http://www.indepth-network.org) based on the World Health Organization VA questionnaire. The questionnaire was adapted to local customs and culture and translated in Bangla by the VA team at the Matlab HDSS. It also includes a structured questionnaire on health care seeking behaviour during the fatal illness episode.

### Data collection

Once the CHRW identified a neonatal death, an interviewer trained in verbal autopsy visited the home 2-6 weeks after the date of death to conduct the interview. After obtaining informed verbal consent, interviews were conducted, generally with the mother, but sometimes with other family members to supplement the interview data. Interviews generally lasted for 40 to 60 minutes depending on the illness history and the emotional state of the caretakers.

### Data analysis

All data were entered via Visual FoxPro data entry screen into the Oracle database. Health care consultations during fatal illness episode were described using simple descriptive statistics. Health services providers were cross-tabulated with sex of deceased newborn, age at death and service area (ICDDR,B maternal and child health project area and adjoining Government health services area). Plurality of treatment visit also was compared by sex of newborn and age at death. Binary logistic regressions were performed to assess the association between the explanatory variables and health care providers among the neonatal death cases. The results are reported with adjusted odds ratios with 95% confidence interval and p-value. Data were analyzed using the *statistical *software Stata version 9 [16].

### Ethical approval

The Human Research Ethics Committee of Curtin University of Technology and the Ethical Review Committee of the International Centre for Diarrhoeal Disease Research, Bangladesh (ICDDR,B) approved the study. Specially trained staff members took verbal consent and conducted the interview at the home of the deceased neonate, respecting standard ethical practice in relation to human research. The purpose of the study was clearly explained and participants were assured that neither participation nor refusal would have any effect on their receipt of standard health care from ICDDRB. No remuneration was offered for participation. Permission was sought to access records on treatment received before death. The issue of right to withdraw was emphasized, with interviews discontinued or resumed at a later stage if the participant requested this. Participants had the right to refuse to answer any question or to withdraw consent at any point without coercion or pressure. All data collected were coded so that participants cannot be identified to maintain the confidentiality. Special care was taken to avoid causing any emotional distress to participants, by using a respectful and compassionate approach during interviewing.

## Results

### Health Care Consultations

Vaginal deliveries at home were the most common form of deliveries in Matlab, although there were significant differences in regards to place and mode of delivery between the two service areas.

Table 1 provides information on neonatal death by place and mode of delivery, as obtained from mothers during the VA. Overall, around 95% of deliveries were vaginal deliveries and five percent (5.2%) of the deliveries required caesarian section. However, the caesarian section rate was much higher in the ICDDR,B area compared to the government area (9.3% versus 1.6%).

**Table 1:** Neonatal deaths by place of delivery, by mode of delivery and by service area

	Government		ICDDR,B		Total	
	n	%	n	%	n	%
Home	161	84.74	88	51.46	249	68.98
Institutional	29	15.26	83	48.54	112	31.02
Total	190	100	171	100	361*	100

Pearson chi2(1) = 46.57 p < .001 * Place of birth information is missing in fours						

Vaginal	189	98.44	157	90.75	346	94.79
Caesarian	3	1.56	16	9.25	19	5.21
Total	192	100	173	100	365	100

Pearson chi2(1) = 10.89 p < .001

Overall, 64% of deliveries were conducted at home. The rate of home delivery was 51% in the ICDDR,B area and 84% in the government service area.

Health care providers consulted by the caregiver of deceased neonates during the fatal illness episode are presented in Table 2. More than a third (38%) of the neonates received no external health care. Medical doctors were consulted in about a quarter of cases (24%), while paramedics treated 13% of cases. Non-medically qualified persons included kabiraj or herbalists who treated 8% of cases, informally trained allopath (8.0%) and homeopaths (6%).

**Table 2:** Health care provider consultation during neonatal fatal illness episodes by Service Area

	ICDDR,B MCH area		Government area		Both	
			
Care provider	N	(%)	N	%	N	%
No treatment	50	(28.9)	87	(45.3)	137	(37.5)
MBBS	60	(34.7)	27	(14.1)	87	(23.8)
Paramedic	35	(20.3)	14	(7.3)	49	(13.4)
Others						
Kabiraj	11	(6.4)	18	(9.4)	29	(8.0)
Unqualified Allopaths	7	(4.1)	22	(11.5)	29	(8.0)
Homeopath	6	(3.5)	15	(7.8)	21	(5.8)
Spiritual Healer	4	(2.3)	7	(3.7)	11	(3.0)
Pharmacy	0	(0)	2	(1.0)	2	(0.6)
**Total**	**173**	**(100)**	**192**	**(100)**	**365**	**(100)**

Pearson Chi2 (3) = 44.73; p < .001

Local pharmacies, which are often little more than a shop front selling medicines by people with no pharmaceutical training were the least utilized source of consultation for neonatal fatal illnesses in both areas.

### Health Care consultation by sex of newborns

Overall, significantly more (p < .01) male newborns were likely to have been taken to a medically qualified source (doctors or paramedics) compared to female newborns. Consultations with non-medically qualified providers (herbalists, unqualified allopath, homeopath, and spiritual healer) were similar for both male and female newborns. Nearly, half of the female newborns (46.7%) did not receive any treatment, compared to 30% of males not receiving any treatment.

### Health Care consultation by early and late neonatal period

The treatment/consultation patterns during the fatal illness episodes varied by age of the neonate. Cases of late neonatal (8-28 days) deaths were more likely to have been seen by both medically qualified providers and non-qualified providers than cases of early neonatal (0-7 days) deaths. A significantly higher (p < .001) proportion (42%) of early neonatal deaths did not receive any treatment compared to 16% of late neonatal deaths.

### Multiple consultations

Around 22 per cent of neonates had multiple consultations during their illness prior to death. Such multiple consultations varied by sex and time of death, as shown in Table 3

**Table 3:** Number of treatment visits according to timing of death and gender

	Number of treatment visits					
		0	1	2	3	>3
						
	N	(%)	(%)	(%)	(%)	(%)
**Sex of newborn**						
Male	200	(30.0)	(45.0)	(19.0)	(5.0)	(1.0)
Female	165	(46.7)	(34.6)	(13.9)	(3.0)	(1.8)
All	365	(37.5)	(40.3)	(16.7)	(4.1)	(1.4)
**Timing of death**						
0-7 days	307	(41.7)	(41.0)	(15.0)	(1.6)	(0.7)
8-28 days	58	(15.5)	(36.2)	(25.9)	(17.2)	(5.2)
**Service area**						
ICDDR,B	173	(28.9)	(48.0)	(17.9)	(3.5)	(1.7)
Government	192	(45.3)	(33.3)	(15.6)	(4.7)	(1.0)
All	365	(37.5)	(40.3)	(16.7)	(4.1)	(1.4)

### Treatment visit by sex of newborn

Multiple consultations were more likely for male newborns than female ones. Second and third consultations were more common for males than for females (19.0% and 5.0% versus 13.9% and 3.0% respectively).

### Treatment visit by early and late neonatal period

The number of consultations varied according to the timing of newborn death. A higher proportion of late neonatal death received second and third consultations compared to early neonatal deaths. The proportion of cases with second and third consultations for late and early neonatal deaths was 25.9% and 17.2% compared to 15.0% and 1.6% respectively.

Male gender, institutional delivery, ICDDRB area and late neonatal death cases were significantly more likely to receive care from a qualified care provider during the fatal illnesses episode. The increased attendance in the ICDDR,B service area suggest that access, including financial costs, and quality of care have a substantial impact on care seeking (Table 4 and Table 5)

**Table 4:** Adjusted odds ratios of covariates for seeking care from any care providers (0 = no care;1 = care from any provider) during fatal neonatal illness

		Neonatal death			p	95% Confidence Interval	
Covariates		N	%	Odds Ratio		Lower	Upper
**Sex of newborn**	Male	200	54.79	1			
	Female	165	45.21	0.43	0.001	0.26	0.71
**Mode of delivery**							
	Vaginal	346	94.79	1.00			
	Caesarian	19	5.21	1.63	0.562	0.31	8.54
**Place of delivery**							
	Home	249	68.98	1.00			
	Institutional	112	31.02	7.60	<.001	3.80	15.21
**Service area**							
	Government	192	52.6	1.00			
	ICDDR,B	173	47.4	1.17	0.545	0.70	1.95
**Timing of death**							
	Early Neonatal	307	84.11	1.00			
	Late Neonatal	58	15.89	7.73	<.001	3.36	17.76

**Table 5:** Adjusted odds ratios of covariates for seeking care from qualified health care providers (0 = no care and care from unqualified provider; 1 = care from qualified care provider) during fatal neonatal illness

		95% Confidence Interval			
		
Covariates		Odds Ratio	p	Lower	Upper
**Sex of newborn**					
	Male	1.00			
	Female	0.48	0.023	0.25	0.90
**Mode of delivery**					
	Vaginal	1.00			
	Caesarian	1.97	0.462	0.32	12.01
**Place of delivery**					
	Home	1.00			
	Institutional	35.96	<.001	17.38	74.38
**Service area**					
	Government	1.00			
	ICDDR,B	2.19	0.013	1.18	4.07
**Timing of death**					
	Early Neonatal	1.00			
	Late neonatal	2.61	0.019	1.17	5.84

## Discussion

The most striking finding of our study was that qualified medical care as the first resort of care was sought in only little more than a third (37%) of the final illness episodes leading to neonatal death in the Matlab HDSS area, while 38% received no external care at all, and the remaining 25% received traditional/unqualified care.

The reasons for the disturbingly high proportion of fatal illness episodes that were not treated by any type of practitioners are similar to reasons reported by other studies from Bangladesh. Reasons identified in these studies include the little time that was available to seek care because of the sudden onset of symptoms, the lack of supportive family members, and cultural restrictions on the mobility of women in public, especially after delivery [5,6]. A recent study reported reasons why parents often did not comply with the advice for referral to a hospital given by local health workers/practitioners. These included lack of supportive family members to accompany the mother, bad weather or political disturbances, family dislike of hospital care, the illness resolving spontaneously, and other reasons including the infant being considered too young to be taken for outside care [17]. Another study conducted during 2002-3 in two rural areas of Bangladesh found that while mothers were generally aware of danger signs of neonatal illnesses, particularly pneumonia and diarrhea, they were less aware of asphyxia and hypothermia as causes of neonatal deaths [3].

The substantial proportion (42%) of early neonatal deaths in our study that did not receive any health care outside the home is of great concern and compares with 16% of late neonatal deaths that did not receive such care. There could be several reasons for this finding, which have also been reported by others [3,18]. First, the cultural tradition of maternal seclusion during the postpartum period is likely to hinder the prompt recognition and treatment of serious neonatal illnesses. Second, a high proportion of neonatal deaths occur very rapidly (within 24 hours) and is another likely reason for parents not seeking health care outside home. Third, around 87 per cent of births in Bangladesh occur at home, limiting access to ready health care at the time when it is needed. Skilled attendance during childbirth and in postnatal care can play a crucial role in preventing neonatal deaths. Progress within Bangladesh in ensuring skilled attendance at delivery has been disappointing, with a recent national survey indicating it had increased from 9% in 1993-94 to only 13% of births in 2004 [10,11]. The survey also found that 83 per cent of newborns received no medical care within 42 days of birth [10].

The reliance on unqualified and or traditional practitioners observed in our study has been reflected in several studies in Bangladesh. A nationwide study of neonatal illnesses found that most parents sought care from unqualified providers such as homeopath (38%) and village doctors (37%). However, mothers who attended antenatal care were more likely to seek care for their newborn's illnesses from a qualified health care provider [5].

A sex differential for seeking care was observed in the present study. Regardless of treatment type, care seeking was greater for males compared with females in the Matlab setting. For nearly half of the female newborns in this study, no care was sought compared to no care sought for 30% of male newborns. Furthermore, male newborns were more likely to have received treatment from a medically qualified source (medical doctors and paramedics) than female newborns. Multiple consultations were also higher for male newborns compared to female. This sex bias has been reported from other studies in Bangladesh [5,19].

The reasons for not seeking care or inappropriate care seeking for neonatal illnesses requires further research. Community awareness about prompt medical care seeking for neonatal illnesses, awareness of the danger signs of serious neonatal illnesses, integration of informal practitioners in public health programmes, and improved access to science-based health care including skilled delivery care and essential newborn care are all strategies that require consideration to reduce neonatal mortality in Matlab and other rural areas in Bangladesh.

Among the neonatal deaths, a relatively higher proportion occurred in institutional delivery cases compared to home delivery cases which possibly indicates that higher proportion of complicated labour cases took place at a facility rather than at home. Another study from Matlab also reported that perinatal deaths were higher among women who sought skilled care than those who did not [20]. These findings suggest that timely and effective obstetric care during delivery as well as early diagnosis of health problems during pregnancy can play an instrumental role to improve the neonatal death. The increased attendance at health services in the ICDDR'B service area suggests that access issues (including concerns about financial cost) have a substantial impact on care seeking, and that improving availability to quality, affordable services will be important for reducing neonatal mortality.

## Limitations

This study collected information on source of care seeking but did not collect much detail on care seeking that limited the conclusions on the circumstances and barriers to care seeking in the study. The study also has the usual drawbacks common in observational studies. Information on certain important confounders could not be collected, and this could have biased the associations detected. For example, the greater association between institutional delivery and neonatal mortality could also have been due to the higher proportion of complicated labour cases that took place at a facility rather than at home. This study reports place of delivery, but did not collect information about the circumstances that proceeded to delivery limiting the conclusion that more complicated deliveries ended up giving birth in health centre. Other known factors such as economic status, which may have influenced the patterns of care seeking detected in the study were not elicited.

## Conclusions

Programme managers can use epidemiological information derived from verbal autopsies to better plan programme interventions. For example, the great majority of neonatal deaths occurred either at the place of delivery or within 7 days of delivery. Universal access to timely and effective obstetric care at delivery especially at a health facility and a postpartum care strategy are key to protecting newborns at their most vulnerable period. Information on health care seeking during the fatal illnesses episode can be usefully adapted to more effectively utilize health care services. Community awareness about prompt care seeking, skilled attendance at delivery and integration of traditional care providers into mainstream health programmes may be an approach to reducing neonatal mortality in developing countries.

Formative research at the local level is needed to understand the use and non-use of facilities for both delivery and newborn care. Findings from such research can help inform approaches for improving early recognition of danger signs, promoting demand for quality of care and overcoming socio-cultural barriers to referral. A greater emphasis on the importance of educating women to recognize signs and symptoms that require medical assessment can be instrumental in further improving neonatal survival. As suboptimal care is a concern in most maternity clinics in developing countries, education of maternity care personnel will benefit from a further focus on how to recognize and manage high-risk pregnancies and newborns. The introduction of regular community death audits or social autopsy could help engaging community in identifying avoidable risk factors for perinatal and neonatal deaths [7,21,22]. Gender discrimination and great poverty coexist in Bangladesh and contribute to a large number of neonatal deaths either through limiting access to effective care or through increasing the magnitude of risk factors such as poor maternal nutrition and health. Addressing such inequity should be a priority of all programme/strategies for improving survival of newborn babies.

## Competing interests

The authors declare that they have no competing interests.

## Authors' contributions

HRC carried out the study design, data management, analysis and drafted the manuscript. NA manages all the data and PKS manages the Matlab HDSS, and participated in the study design and revising the manuscript. SCT and MA provided help with the study design, data analysis, write-up and editing of the manuscript, while MY contributed in the study design, read the manuscript critically and helped with revising the manuscript. All the authors approved the final version of the manuscript.

## Pre-publication history

The pre-publication history for this paper can be accessed here:

http://www.biomedcentral.com/1471-2431/11/88/prepub
